# Low fish oil intake improves insulin sensitivity, lipid profile and muscle metabolism on insulin resistant MSG-obese rats

**DOI:** 10.1186/1476-511X-10-66

**Published:** 2011-04-28

**Authors:** Ricardo K Yamazaki, Gleisson AP Brito, Isabela Coelho, Danielle CT Pequitto, Adriana A Yamaguchi, Gina Borghetti, Dalton Luiz Schiessel, Marcelo Kryczyk, Juliano Machado, Ricelli ER Rocha, Julia Aikawa, Fabiola Iagher, Katya Naliwaiko, Ricardo A Tanhoffer, Everson A Nunes, Luiz Claudio Fernandes

**Affiliations:** 1 Department of Physiology, Biological Sciences Building, Federal University of Parana, Curitiba-PR, Brazil; 2 Physiological Sciences Department, Biological Sciences Center, Federal University of Santa Catarina, Florianopolis-SC, Brazil

## Abstract

**Background:**

Obesity is commonly associated with diabetes, cardiovascular diseases and cancer. The purpose of this study was to determinate the effect of a lower dose of fish oil supplementation on insulin sensitivity, lipid profile, and muscle metabolism in obese rats.

**Methods:**

Monosodium glutamate (MSG) (4 mg/g body weight) was injected in neonatal Wistar male rats. Three-month-old rats were divided in normal-weight control group (C), coconut fat-treated normal weight group (CO), fish oil-treated normal weight group (FO), obese control group (Ob), coconut fat-treated obese group (ObCO) and fish oil-treated obese group (ObFO). Obese insulin-resistant rats were supplemented with fish oil or coconut fat (1 g/kg/day) for 4 weeks. Insulin sensitivity, fasting blood biochemicals parameters, and skeletal muscle glucose metabolism were analyzed.

**Results:**

Obese animals (Ob) presented higher Index Lee and 2.5 fold epididymal and retroperitoneal adipose tissue than C. Insulin sensitivity test (Kitt) showed that fish oil supplementation was able to maintain insulin sensitivity of obese rats (ObFO) similar to C. There were no changes in glucose and HDL-cholesterol levels amongst groups. Yet, ObFO revealed lower levels of total cholesterol (TC; 30%) and triacylglycerol (TG; 33%) compared to Ob. Finally, since exposed to insulin, ObFO skeletal muscle revealed an increase of 10% in lactate production, 38% in glycogen synthesis and 39% in oxidation of glucose compared to Ob.

**Conclusions:**

Low dose of fish oil supplementation (1 g/kg/day) was able to reduce TC and TG levels, in addition to improved systemic and muscle insulin sensitivity. These results lend credence to the benefits of n-3 fatty acids upon the deleterious effects of insulin resistance mechanisms.

## Background

The number of obese adults and children around the world has grown dramatically in the past years. This is a major concern for public health, since, obesity is commonly associated to other conditions such as cardiovascular disease (CVD), type 2 diabetes and some types of cancer [1]. Physical inactivity and high-fat diets [2], may be considered the main causes for the increased incidence of overweight and obesity. In fact, obesity has been strongly associated with metabolic syndrome (MS), which refers to a clustering of CVD risk factors including insulin resistance, type 2 diabetes, dyslipidaemia and hypertension [3].

The obesity model induced by monosodium glutamate (MSG) has demonstrated some of the clinical features observed in individuals with MS [4]. The monosodium glutamate injected subcutaneously in neonatal period causes hypothalamic damage [5], and as a consequence, these animals present several neuroendocrine and metabolic alterations, which leads to higher levels of adipose tissue accumulation, insulin resistance and hyperinsulinemia [6]. So the use of this model may add new information concerning the effects of possible treatments for both obesity and risk factor for CVD commonly observed in MS.

Polyunsaturated fatty acids such as n-6 is largely available in high-fat diets, and epidemiological studies have shown that increased ratio of n-6 to n-3 fatty acids in diet has been proportionally related to increased incidence of type 2 diabetes, CVD, and inflammatory diseases [7,8]. Yet, recent evidence has also linked intake of saturated fats (i.e. coconut fat), with development of obesity [9]. Conversely, dietary intake of fish oil, which presents high amounts of n-3 fatty acids such as eicosapentaenoic acid (EPA) and docosahexaenoic acid (DHA), has presented beneficial effects on diabetes and obesity [10]. Several studies have examined the effects of n-3 PUFA on the prevention of insulin resistance [11,12], however, few data have shown improvement in insulin resistance conditions owing to dietary fish intake [13]. In addition, the concentration of fish oil used in most studies are high (7 wt% or more) and further future studies are important to determine the efficacy of lower concentrations of fish oil. Although the effects of fish oil supplementation on insulin resistance and dyslipidemia have been previously reported, results in this area are still inconclusive. Therefore, the aim of this study was to investigate the effects of lower concentration of fish oil supplementation on fasting lipid profile, insulin resistance and skeletal muscle glucose metabolism of obese rats induced by monosodium glutamate.

## Results

### Obesity parameters and insulin sensitivity

Table 1 summarizes the lipid profile from standard diet and oils. These results confirm the high amounts of n-3 polyunsaturated fatty acids EPA and DHA on fish oil, while the coconut fat has presented mainly saturated fatty acids (lauric, myristic and palmitic). Table 2 summarizes some of the characteristics of the MSG model. MSG-treated rats became obese as confirmed by higher Index Lee, even without hyperphagia. Table 2 also shows that, although the obese group did not gain more weight, they presented higher epididymal and retroperitoneal fat deposits, which is a central characteristic of obesity status.

**Table 1:** Fatty acid composition of diet and oils

Fatty acids (g/100 g of total fatty acids)	Standard Diet	Fish Oil	Coconut Fat
12:0	1.2 ± 0.05	5.8 ± 0.6	30.8 ± 1.1
14:0	-	-	16.1 ± 0.2
16:0	22.24 ± 2.6	20.9 ± 1.3	48.9 ± 1.1
16:1 n-7	-	-	-
18:0	3.1 ± 0.3	1.8 ± 0.3	1.9 ± 0.2
18:1 n-9	17.3 ± 0.4	9.1 ± 0.5	7.9 ± 0.2
18:2n-6	51.6 ± 2.4	2.4 ± 0.6	2.0 ± 0.2
18:3n-3	5.3 ± 0.5	-	0.9 ± 0.01
20:4n-6	0.2 ± 0.05	9.7 ± 0.1	-
20:5n-3	0.2 ± 0.04	20.9 ± 1.2	0.7 ± 0.06
22:6n-3	-	26.35 ± 3.1	-

Summary of fatty acids profile of diet and oils.

**Table 2:** Morphometric parameters

Group	Lee index	Weight (g)	Food consumption (g/100 g)	Epididymal AT (g/100 g)	Retroperitoneal AT (g/100 g)
**C**	314.00 ± 2.89	367.50 ± 7.50	7.27 ± 0.11	0.85 ± 0.06	0.96 ± 0.10
**FO**	314.7 ± 2.44	371.70 ± 6.70	7.03 ± 0.11	1.00 ± 0.05	1.10 ± 0.07
**CO**	313.00 ± 2.17	375.00 ± 6.31	7.18 ± 0.15	0.89 ± 0.06	1.02 ± 0.09
**Ob**	326.60 ± 4.19*	311.00 ± 8.14*	7.16 ± 0.19	2.06 ± 0.15*	2.45 ± 0.12*
**ObFO**	332.00 ± 3.87	310.00 ± 10.19	6.73 ± 0.16	2.02 ± 0.12	2.50 ± 0.13
**ObCO**	325.40 ± 2.74	312.90 ± 7.10	6.96 ± 0.18	2.13 ± 0.13	2.70 ± 0.14

Animals with 120 days of age. * when compared to control (C) group

**Figure 1:**
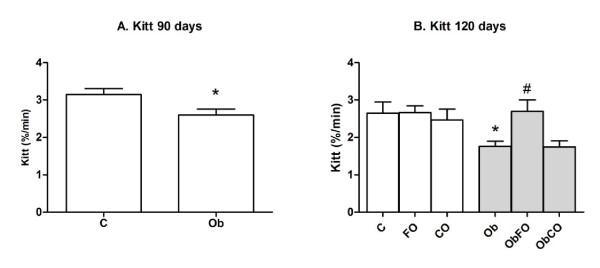
**Insulin sensitivity**. A. Kitt from animals with 90 days of age. B. Kitt from animals with 120 days of age. * when compared to C group; # when compared with Ob group.

Obese animals with 90 days of age were insulin resistant as confirmed by insulin tolerance test (Kitt) (p < 0.05) (Figure 1A). The fish oil supplementation was able to reverse this parameter by showing insulin sensitivity similar to healthy animals (p > 0.05) (Figure 1B).

### Lipid profile

**Figure 2:**
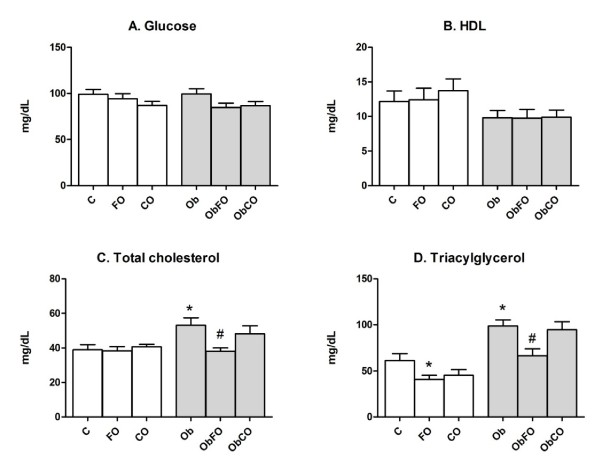
**Biochemical plasma parameters**. A. Glucose; B. HDL - cholesterol; C. Total cholesterol; D. Triacylglycerol. * when compared with C group; # when compared with Ob group

No significant difference amongst all groups was found in glycemy and HDL levels (Figures 2A and 2B). TC levels were high on Ob group (p < 0.05) (Figure 2C). Fish oil supplementation reduced TC levels of obese animals by 30% (p < 0.05; ObFO vs. Ob) while coconut fat supplementation did not promote any effect. TG levels were reduced by 34% and 33% in the obese (ObFO) and healthy (FO) fish oil supplemented rats when compared to their respective control groups (Ob and C) (Figure 2D).

### Muscle metabolism

**Figure 3:**
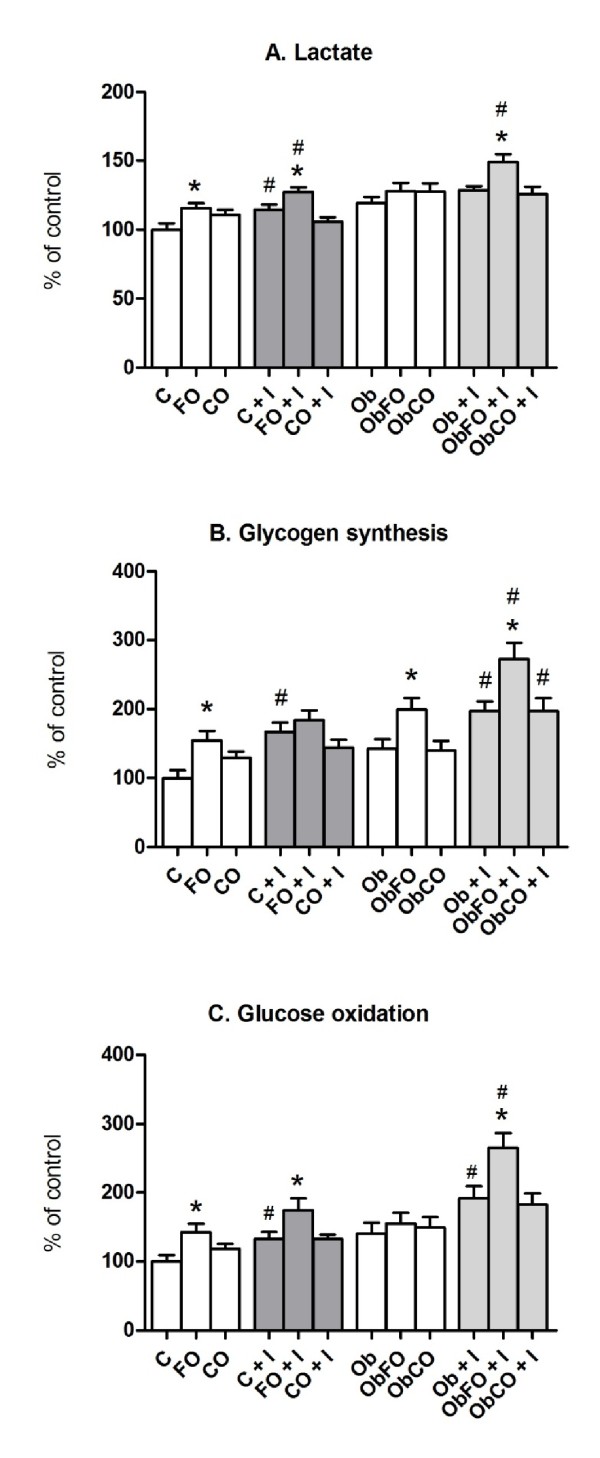
**Muscle metabolism**. A. Lactate production by skeletal muscle incubated; B. Glycogen synthesis from incubated skeletal muscle; C. Glucose oxidation from skeletal muscle incubated. 100% was considered as the median of the values obtained in control group. * when compared with their respective control group; # when compared with the respective group without stimuli.

Incubated skeletal muscle from FO group had 15% higher lactate production when compared to C group (p < 0.05) (Figure 3A). Coconut fat supplementation did not alter lactate production by skeletal muscle when compared to control group (p > 0.05). Insulin stimuli increased lactate production by 14% on control group and by 10% on FO group when compared with the absence of stimulus (p < 0.05). No alteration was observed in ObCO after added insulin. Interestingly, only the ObFO group plus insulin presented an increase of 10% when compared with absence of insulin (p < 0.05). In presence of insulin, C and CO did not alter lactate production when compared with their respective control groups (p > 0.05).

FO group revealed increased skeletal muscle glycogen synthesis by 50% without insulin stimulus (p < 0.05) (Figure 3B). In obese group without supplementation (Ob), the glycogen synthesis was not different when compared to C group. ObFO presented a 40% increase in glycogen synthesis when compared to Ob group (p < 0.05). Insulin increased glycogen synthesis by 66% in C group (p < 0.05). Nevertheless, the basal condition on glycogen synthesis was high in the FO group and no additional effect was observed in the presence of insulin (p > 0.05). In CO plus insulin group, synthesis of glycogen was similar to basal conditions. The Ob group plus insulin increased the glycogen synthesis by 38% when compared to basal conditions (p < 0.05). On supplemented groups (ObFO and ObCO) plus insulin, fish oil and coconut fat increased by 36% and 45%, respectively, when compared to absence of stimuli (p < 0.05).

FO increased the glucose oxidation of skeletal muscle by 42% in basal condition when compared to control group (p < 0.05) (Figure 3C). CO did not cause any effect, as well as the obesity condition supplemented or not (p > 0.05). Insulin increased the glucose oxidation by 32% on C group when compared to basal condition (p < 0.05). FO also increased by 31% glucose oxidation in the presence of insulin (p < 0.05) when compared to C+I group, while coconut fat supplementation did not cause any effect. In Ob group plus insulin, glucose oxidation was increased by 45% when compared to basal conditions (p < 0.05). ObFO plus insulin also increased by 71% when compared to basal conditions (p < 0.05).

## Discussion

The obese animals presented higher Lee index and increase of fat depots (Table 2). These results confirm the obesity induction by glutamate monosodium and corroborate previous studies [14,15]. Several metabolic changes of this model occur due to hypothalamic lesions, mainly on the arcuate nucleus [16]. These lesions result in impaired insulin signaling and reduction of GHRH (Growth Hormone Releasing Hormone), which is associated with body length reduction also related to reduction of body weight (Table 2). This model also presents reduction of sympathetic activity and low HSL (Hormone Senstive Lipase) activity which is an enzyme with a role in the triacyglycerol hydrolysis [17]. These alterations in adipose tissue metabolism may explain the high quantities found in epididymal and retroperitoneal adipose tissues (Table 2). Thus, the MSG rats have been used for studies of obesity because they usually present insulin resistance and high depots of fat [17-21]. Interestingly, there are no reports on employing this model to examine the effects of fish oil supplementation.

Rats fed under an isocaloric high fat diet plus 7% of fish oil showed improved insulin sensitivity quantified by euglycemic-hyperinsulinemic glucose clamp [22]. Andersen and coworkers showed reduction of glycemy and improved insulin sensitivity by HOMA test after oral supplementation of EPA or DHA (0.5 g/kg) for 8 weeks [12]. Our study corroborates these previous finding, showing the ability of fish oil supplementation to improve insulin sensitivity in obese animals. In contrast, Gillam and coworkers did not find any effect of the fish oil (10%) added to diet on insulin resistance, pancreatic function and glucose metabolism [23]. The discrepancy between these results may be due the concentration of fish oil added to diet and models used, i.e., high fat and/or high-sucrose diets to induce obesity, or genetically obesity models.

Dyslipidaemia is a common feature in diabetic people and it is strongly associated with the development of atherosclerosis [24]. The MSG rats presented higher levels of TG and cholesterol, as previously described [21]. Lipoproteins rich in TG play a role in inflammatory process through NF-κB activation [25]. Thus, the reduction of TG levels found within this study and others [26,27] is an important factor which might explain part of the beneficial effects of n-3 fatty acids on cardiovascular diseases. Hypercholesterolemia increases the expression of adhesion molecules involved in atherogenesis [28]. Our study also showed the ability of fish oil to reduce cholesterol levels of obese rats.

Skeletal muscle is the principal site of glucose metabolism and represents 40 to 50% of the total body mass. Nevertheless, to best of our knowledge, there is no report about the changes on muscle metabolism of the MSG rats. Analyzing the results from muscle metabolism responses and comparing them to systemic insulin resistance, data might seem paradoxal. There are possible reasons for these findings. First, the results from systemic insulin resistance may be influenced by several factors, besides the muscle, such as free fatty acids [29], cytokines [30] and other insulin targets like adipose tissue and liver [31]. Second, a state of hyperinsulinemia usually found in this model could result in the increase of cell metabolism for a short period [32]. D'Alessandro and coworkers showed that fed rats with a diet plus 7% fish oil increased insulin sensitivity of skeletal muscle [33]. This study also found that under insulin stimuli, the glycolytic pathway was more activated rather than oxidation and storage components of glucose metabolism. This result differs from the data herein reported, probably owing to different deployed methodologies i.e. muscle type and enzymes analyzed. Interestingly, our study is the first to report these effects using a lower dose (1 g/kg/day) orally administrated on obese rats induced by MSG.

## Conclusions

In conclusion, fish oil supplementation was able to reduce triacylglycerols and cholesterol levels, in addition to improvement in systemic and muscle insulin sensitivity. These results show that n-3 fatty acids might have crucial role in preventing and reversing insulin restance.

## Methods

### Materials

All enzymes and reagents for buffers were obtained from Sigma Chemical (St. Louis, MO). Fish oil was purchased from Herbarium (Parana, Brazil), coconut fat from Refino de Oleos (Bahia, Brazil), and standard chow (Nuvilab CR-1) diet from Nuvital Nutrientes (Curitiba, Brazil). Macronutrients of standard chow presented 63.4% carbohydrates, 25.6% of proteins and 11% of lipids. Fatty acid composition of the oils and chow was determined by high-performance liquid chromatographer as described previously [34]. Briefly, total lipids were extracted from oils and chow using chloroform-methanol (2:1 vol/vol) according to Folch *et al*. [35] and free fatty acids were obtained by saponification. Fatty acids were than derivatized with 4-bromomethyl-7-coumarin and then separated by high performance liquid chromatograph (Varian ProStar) using an octadecylsilica column (25 cm × 4, 6 mm i.d.; particle size 5 mm). Fatty acids were resolved isocratically using a mobile phase of acetonitrile-water (gradient from 77:23 to 90:10 vol/vol) and fatty acid derivatives were detected by fluorescence (325-nm excitation; 395-nm emission).

### Animal model and supplementation

Wistar rats were provided by the Experimental Breeding Center of the Federal University of Parana. All animal procedures were approved by the Ethical Committee of Animal Research, Sector of Biological Sciences, Federal University of Parana. Monosodium glutamate (MSG) (4 mg/g body weight) was subcutaneously injected during the first 5 days after birth. Control animals received equimolar solution of saline. On the 21^st ^day, animals were weaned and housed under control conditions, in a 12-h light-dark inverted cycle (10 am. to 10 pm.) and 22 ± 2°C temperature. Water and standard chow were supplied *ad libitum*. At 90 days of age, animals were anesthetized with penthobarbital (50 mg/kg) followed by intravenous insulin test to confirm insulin resistance. After, groups were divided in control (C), fish oil supplemented (FO), coconut fat supplemented (CO), obese (Ob), obese fish oil supplemented (ObFO) and obese coconut fat supplemented (ObCO). Supplemented groups received oral administration provided as a single bolus daily using a micropipette of fish oil or coconut fat (1 g/kg/day) for 4 weeks.

Body weight and food intake were recorded every other day. At the end of the supplementation period, another intravenous insulin test was performed. After an overnight fasting, animals were anesthetized and killed by decapitation. After centrifugation, plasma samples were assayed for analysis of plasma biochemicals. Tissue samples were collected and kept at -80°C for subsequent analysis.

### Index Lee

The index Lee [body weight (g)^1/3 ^by the naso-anal length (cm) × 1000] was used as a parameter to evaluate the degree of obesity [36]

### Insulin tolerance test

For estimation of *in vivo *insulin sensitivity, insulin was injected intravenously (0.75 U/kg body weight) in 12 h-fasted obese and non-obese rats. Afterwards, tail blood samples were collected at 0, 4, 8, 12 and 16 minutes. The rate for blood glucose disappearance was calculated based on the linear regression of the blood glucose concentrations obtained from 0 to 16 min of the test [37].

### Analysis of biochemical plasma parameters

Enzymatic colorimetric assay kits adapted for a microplate reader (Infinite 200 TECAN) were used to determine fasting plasma glucose, triacylglycerol and total cholesterol. Enzyme immunoassay kit (SPI-Bio Bertin Pharma, Montigny le Bretonneux, France) was used to measure fasting plasma insulin.

### Lactate production by incubated tissue

Under penthobarbital anesthesia (50 mg/kg), rats were killed by cervical dislocation, and soleus muscles were isolated, split longitudinally in portions weighing 20 to 30 mg. The muscles were preincubated in a water-bath, gently agitated, for 30 min at 37°C in Krebs-Ringer (KR) bicarbonate buffer, pH 7.4, pregassed for 30 min with 95% O_2 _- 5% CO_2_, containing 5.6 mM glucose and 1% BSA. After preincubation, muscles were transferred to the same buffer under similar conditions for 1 hour, in the absence or presence of 10 mU/mL insulin. At the end of incubation period, lactate from the medium was determined as previously described [38].

### D-[U-^14^C] glucose incorporation to glycogen and oxidation to CO_2 _by incubated skeletal muscle

Following the same incubation procedures, muscles were transferred to other vials containing the same buffer, but added 0.3 Ci/mL D-[U-^14^C] glucose. One hour incubation was performed in the absence or presence of insulin (10 mU/mL) to the KR buffer, muscles were digested in KOH solution and glycogen synthesis determined as previously described. Phenylethylamine, diluted 1:1 v/v in methanol, was added into a separate compartment for ^14^CO_2 _adsortion and D-[U-^14^C] glucose oxidation. [^14^C]-glycogen synthesis (estimated by [D-^14^C]-glucose incorporation into glycogen) was determined as described by Espinal *et al *[39].

### Statistical Analysis

Data was tested for normal distribution with D'Agostino-Pearson test and differences between groups were analysed using unpaired t-test and one-way analysis of variance (ANOVA) followed by post hoc Tukey test. A value of p < 0.05 was taken to indicate statistical significance (Graphpad PRISM). All results are expressed as the mean ± standard error mean (SEM).

## Competing interests

The authors declare that they have no competing interests.

## Authors' contributions

RKY wrote the manuscript. RKY, GAPB, IC, DCTP, AAY, GB, DLS, MK, JM, RERR, JA, FI, KN and EAN conducted data collection and analysis. RKY, GAPB, IC, DCTP, RAT, EAN and LCF were also involved on the review and editition of the manuscript. All authors made critical comments during study design and preparation of manuscript and have given their final approval of the version to be published.
